# Patient factors and outcomes associated with discordance between quantitative and qualitative cardiac PET ischemia information

**DOI:** 10.1371/journal.pone.0246149

**Published:** 2021-03-03

**Authors:** Haley Zigray, Shana Elman, Richard K. Cheng, Song Li, James Lee, Laurie Soine, James Caldwell, Adam M. Alessio

**Affiliations:** 1 School of Medicine, University of Washington, Seattle, Washington, United States of America; 2 Department of Radiology, University of Washington, Seattle, Washington, United States of America; 3 Division of Cardiology, Department of Medicine, University of Washington, Seattle, Washington, United States of America; 4 Department of Computational Mathematics, Science, and Engineering, Biomedical Engineering and Radiology, Michigan State University, East Lansing, Michigan, United States of America; University of Dundee, UNITED KINGDOM

## Abstract

**Background:**

Cardiac PET can provide quantitative myocardial blood flow (MBF) estimates. The frequency and clinical significance of discordant ischemia information between quantitative and qualitative parameters is unclear.

**Methods:**

This retrospective, cohort study analyzed 256 Rb-82 stress-rest PET/CT studies. Global MBF and myocardial flow reserve (MFR) were estimated in absolute units for quantitative results and sum-stress and difference scores were used for qualitative results. Four groups of patients were evaluated based on a specific definition of concordant and discordant quantitative and qualitative results.

**Results:**

31% of cases demonstrated discordance. Factors associated with microvascular disease were more common in the groups with abnormal quantitative results, regardless of the qualitative findings. Patients with concordant abnormal results had a significantly increased risk of myocardial infarction, heart failure, percutaneous intervention, and all-cause-mortality at 1 year compared to patients with concordant normal results. In patients with discordant results of abnormal quantitative and normal qualitative findings, there was a higher prevalence of heart failure than in controls (12.5% vs 0%, p = 0.01).

**Conclusions:**

Discordance in qualitative and quantitative ischemia measures from PET is common, and further study is needed to clarify its prognostic implications. Moreover, quantitative estimation of MBF and MFR appears to add value to qualitative visual interpretation by supporting qualitative findings when results are concordant. Abnormal quantitative findings, regardless of concordance or discordance with qualitative findings, occurred in patients with risk factors associated with diffuse disease and with increased risk of heart failure admission.

## Introduction

Nuclear myocardial perfusion imaging (MPI) is most commonly performed using single photon emission computed tomography (SPECT) due to its wide accessibility and high sensitivity for focal epicardial disease [1]. Comparatively, positron emission tomography (PET) has improved spatial resolution and most scanners offer rapid attenuation correction with built in computed tomography (CT) integration. Further, lower doses of radiation, superior diagnostic accuracy, improved sensitivity in diffuse coronary artery disease, and the capability of quantifying myocardial blood flow in absolute units has allowed cardiac PET to emerge as an attractive alternative to SPECT, limited primarily by cost and availability [1–3].

Myocardial perfusion imaging with PET can provide a noninvasive measure of myocardial blood flow (MBF) and myocardial flow reserve (MFR) [4]. In clinical practice, perfusion defects detected with MPI are typically attributed to epicardial coronary artery stenosis. However, this simplification ignores the effect of microvascular disease in several cohorts (including those diabetes, hypertension, heart transplant, and/or tobacco use) [5–8]. Although there is currently no widely-available clinical test to evaluate and distinguish microvascular disease from epicardial coronary artery disease, a growing body of literature is supportive of quantitative cardiac PET for this application.

Quantification of myocardial blood flow with PET and its potential to distinguish between epicardial coronary disease and microvascular coronary disease has many clinical implications. Cardiac PET can be used to identify patients who may benefit from percutaneous coronary intervention (PCI) with good correlation with invasive catheter-based fractional flow reserve (FFR) [9]. Also, serial myocardial perfusion PET can track the response of cardiovascular patients to medical therapy [4, 10–13]. Further, myocardial perfusion PET has prognostic value for predicting major adverse cardiac events (MACE) in patients with microvascular disease [14–18]. Lastly, MFR holds the promise of risk-stratifying the challenging subset of patients who have angina-type symptoms without epicardial coronary artery disease or who have persistent angina after revascularization [19].

While quantification of MBF has proven value, obtaining quantitative MBF results requires additional expense in the form of specialized software, potentially different acquisition protocols, processing/MBF estimation effort, and additional interpretation time. In practice, if the quantitative MBF information is discordant with conventional qualitative findings, this can lead to a lack of clinical adoption of the more expensive quantitative information. The significance and etiology of discordance has not been well studied, and this can pose problems when attempting to devise a treatment strategy for patients with normal qualitative results but abnormal quantitative MFR, or vice versa. The aim of this study was to determine the prevalence of discordant findings from global summary measures of the myocardium and to elucidate some of the patient characteristics and clinical implications associated with discordant PET findings.

## Methods

This minimal risk study was approved by the University of Washington IRB. All data were fully anonymized prior to analysis and the IRB committee waived the requirement for informed consent. Consecutive Rb-82 stress-rest PET/CT exams for the assessment of myocardial ischemia at the University of Washington Medical Center (Seattle, WA) between March 2014 and December 2016 were included in this retrospective, cohort analysis. Patients less than or equal to 21 years of age were excluded. There were 356 exams on patients older than 21 years during this period. Of these, duplicate exams (n = 18) or incomplete imaging exams (n = 17 missing qualitative SDS; n = 65 missing quantitative MBF & MFR) were excluded. The reason for incomplete exams was not documented in the medical record, although likely due to operator error in recording results in the system and/or error during acquisition. Of the remaining set, 15 patients were deemed to have infarcted scar tissue based on imaging criteria discussed below and removed from the subsequent analysis designed to evaluate discordance for ischemia only (in absence of infarction). After this data curation, there were 241 exams.

### PET imaging

Patients were instructed to refrain from caffeine 12 hours, fast for 6 hours, and have no tobacco at least 4 hours prior to exam. Beta blockers and nitrates were held the morning of exam. All imaging was performed in 2D mode on a GE Discovery STE system [20]. At the beginning of the imaging study, a low-dose free breathing cine CT acquisition was performed for attenuation correction acquiring 6 consecutive CT images over 4.8 seconds at each bed location with 120 kVp, 12 mAs each to image patient respiration. These images were averaged to form attenuation correction maps.

The resting exam was performed during injection of Rb-82 at a rate of 50 ml/min. The activity of the injection was based on patient weight with 1110 MBq for patients ≤70 kg, 1480 MBq for patients between 70 kg and 104 kg, and 1850 MBq for patients >104 kg. At start of injection, a dynamic PET acquisition was acquired with frames: 15 x 8s, 5 x 12s, 2 x 30s, 2 x 60s, and 1 x 120s for a total of 8 minutes.

Following an approximately 10-minute delay to allow complete Rb-82 decay, pharmacologic stress was performed with a bolus injection of a unit dose of regadenoson (0.4 mg in 5mL syringe, Astellas, Tokyo) and saline flush. After 1 minute 15 seconds, the stress Rb82 injection and acquisition was performed with the same dosage and frame rate as the resting study. After the stress exam, an additional CINE CT acquisition was performed for attenuation correction following the technique discussed above.

The static and gated cardiac PET images for qualitative image interpretation were formed with the final five and a half minutes of the rest and stress acquisition. These images were reconstructed using the vendor provided OSEM method with 3 iterations, 28 subsets, and a 10mm Gaussian post filter. The dynamic PET images were reconstructed using OSEM with 28 subsets, 6 iterations, and a 5 mm Gaussian post filter.

### Image interpretation

#### Qualitative PET static image interpretation

The qualitative analysis of cardiac PET was based on the visual assessment of differential radiotracer uptake at rest and following vasodilator stress. A semi-quantitative scoring system that uses a 17-segment cardiac model was employed. This analysis involves visual assessment of each segment and attribution of a score on a 5-point scale (where 0 = no defect and 4 = absent radiotracer uptake). The sum of these scores at stress (SSS), rest (SRS), and the difference of these values, summed difference score (SDS), were calculated [21]. All images were reviewed by a board-certified nuclear medicine practitioner and the global SSS, SRS, and SDS were recorded. All patients with primarily infarcted tissue were excluded, defined as having an SDS less than 2 and an SSS greater than 7 [22]. The remaining patients were graded as having an abnormal study with moderate to severe ischemia via qualitative interpretation if the SSS was greater than 7 [22, 23].

#### Quantitative PET dynamic image interpretation

Data were analyzed on our quantitative analysis system (UW-QPP) which was previously validated for NH_3_ and Rb-82 studies [24] and shown by Nesterov et al to provide very similar results to other Rb-82 quantitative packages [25]. Absolute Myocardial blood flow (MBF), flow per unit mass (in mL/min/g) [26], was quantified with an axially distributed model [25] and global rest MBF, stress MBF, and myocardial flow reserve (MFR) [27], ratio stress to rest MBF, were recorded for all exams. Healthy coronary vasculature should augment flow by at least 2-fold during vasodilator stress to meet increased myocardial oxygen demand, and most healthy controls have a MFR well above 2 [11]. A scan was considered to be abnormal via quantitative interpretation if the MFR was less than 2.03 and the stress flow was less than 1.12 ml/g/min, leading to the category of having moderate to severe ischemia according to Johnson and Gould [23].

### Patient characteristics and outcomes

Data regarding patient demographics, clinical characteristics, and hemodynamics were recorded at the time of cardiac PET imaging. Major adverse cardiac event (MACE) data were gathered from the UWMC medical record. This includes revascularization with PCI or coronary artery bypass graft (CABG), hospital admission for myocardial infarction (MI), hospital admission for heart failure (HF), and all-cause mortality. Outcomes were collected from the date of scan to one-year post-scan.

### Patient groups

Patients were divided based on qualitative and quantitative findings for ischemia into 4 groups. Abnormal qualitative ischemia findings were defined as a SSS > 7 and abnormal quantitation as MFR < 2.03 and Stress MBF < 1.12 ml/g/min [23], which are categorizations suggestive of moderate to severe ischemia.

- Group 1 patients with normal qualitative and quantitative results were used as the control.
- Group 2 patients with abnormal qualitative and quantitative results have results commonly associated with ischemia from focal epicardial coronary artery disease.
- Group 3 patients with abnormal qualitative and normal quantitative results have discordance in findings which may be representative of nonpathological flow heterogeneity [28].
- Group 4 patients with normal qualitative and abnormal quantitative results have discordance which may be due to microvascular disease or three-vessel disease.

### Statistical analysis

Patient demographics, clinical characteristics, hemodynamics, and MACE at 1-year were evaluated across groups. Continuous variables were assessed for normality and are displayed as means with standard deviations, and categorical variables are displayed as percentages. Differences between groups were analyzed using T-tests (without assuming equal variances) for continuous variables and F-tests for categorical variables. All p-values <0.05 were considered significant. Cumulative incidence curves were generated for each of the major adverse cardiac events, and Cox proportional hazards models were used for time-to-event analysis for each MACE outcome except MI admission (unable to be performed as no MI admission events in Group 1 were observed to compare the three other groups against).

## Results

A total of 241 Rb-82 stress-rest PET/CT scans were included in the analyses. The average age was 66.0±13.2 years and the average BMI was 29.7±6.7 kg/m^2^. Overall, 69 (28.6%) patients were female, 186 (77%) had hypertension, 90 (37%) had diabetes, and 25 (10%) were smokers. Overall, 190 out of 241 (79%) patients were followed for MACE up to 1 year, and the median follow-up time was 365 days (interquartile range [365,365]).

### Group comparison

Patients were divided into 1 control group and 3 test groups based on concordance or discordance in qualitative and quantitative cardiac PET/CT results: Group 1-Control (Nl-Qual-Nl-Quant, n = 128); Group 2-Abnl-Qual-Abnl-Quant, n = 38; Group 3-Abnl-Qual-Nl-Quant, n = 59; Group 4-Nl-Qual-Abnl-Quant, n = 16. S1 Fig depicts the stratification of patients based on SSS and MFR. Of the total 241 cases, 75 (31%) had discordant results.

Table 1 depicts averages and frequencies for demographics, clinical characteristics, and hemodynamics based on patient group. In analysis of clinical characteristics, both Group 2-Abnl-Qual-Abnl-Quant and Group 4-Nl-Qual-Abnl-Quant had significantly higher frequencies of diabetes (58% and 69% respectively) compared to Group 1-control (30%). The frequencies of hypertension and smoking did not differ from control for any of the three test groups. Analysis revealed that Group 2-Abnl-Qual-Abnl-Quant and 4 had significantly higher average age than Group 1-control (Group 1-control: 64, Group 2-Abnl-Qual-Abnl-Quant: 70; Group 3-Abnl-Qual-Nl-Quant: 68: Group 4-Nl-Qual-Abnl-Quant: 70 years). While not reaching statistical significance and Group 2-Abnl-Qual-Abnl-Quant and 4 had showed a higher average BMI (31 and 32 kg/m^2^) when compared to Group 1-control (29 kg/m^2^).

**Table 1:** Summary of patient demographics and imaging findings clustered by normal and abnormal qualitative and quantitative results. (Online table).

		Concordant Groups		Discordant Groups	
	Total	Group 1 (Nl Qual/Nl Quant)	Group 2 (Abn Qual/Abn Quant)	Group 3 (Abn Qual/Nl Quant)	Group 4 (Nl Qual/Abn Quant)
Number of patients	241	128	38	59	16
Age (years)	66.0 +/- 13.2	63.6 +/- 14.2^	69.6 +/- 9.8*	67.6 +/- 13.5	70.1 +/- 6.8*
BMI (kg/m^2)	29.7 +/- 6.7	29.4 +/- 7.1	30.6 +/- 5.6	28.8 +/- 6.1	32.4 +/- 8.3
Female (%)	28.6	38.3^	15.8*	22.0*	6.2*
Hypertension (%)	77.2	75	78.9	79.7	81.2
Diabetes (%)	37.3	30.5^	57.9*	30.5^	68.8*
Smoker (%)	10.4	12.5	15.8	5.1	0
Prior Cath (%)	81.7	72.7^	97.4*	91.5*	81.2
Prior Heart Tx (%)	14.9	20.3^	5.3*	13.6	0
Peak heart rate (bpm)	92.5 +/- 16.9	95.4 +/- 17.1^	82.9 +/- 11.5*	94.0 +/- 16.8^	86.6 +/- 17.1
Resting heart rate (bpm)	73.5 +/- 13.0	74.8 +/- 12.3^	69.2 +/- 12.4*	73.5 +/- 14.3	73.2 +/- 13.1
Global wall motion abnormality at rest (%)	37.3	14.8^	83.8*	51.7*^	50.0*^
RPP during stress (mmHg/min)	11940 +/- 3297	12381 +/- 3375^	10353 +/- 2457*	12131 +/- 3471^	11351 +/- 2676
RPP at rest (mmHg/min)	9693 +/- 2355	9865 +/- 2251^	8946 +/- 2321*	9891 +/- 2557	9337 +/- 2306
Percent change in RPP, from rest to stress (%)	25.7 +/- 32.4	28.4 +/- 36.4	19.4 +/- 29.1	24.7 +/- 27.7	23.3 +/- 13.5
Ejection fraction at rest (%)	50.8 +/- 15.7	58.1 +/- 11.9^	34.7 +/- 12.6*	47.9 +/- 14.6*^	42.6 +/- 17.9*
End diastolic volume at rest (mL)	110.2 +/- 60.0	84.6 +/- 38.6^	168.4 +/- 69.5*	116.3 +/- 58.9*^	147.8 +/- 58.8*
End systolic volume at rest (mL)	61.1 +/- 51.1	39.1 +/- 31.5^	108.5 +/- 51.7*	67.0 +/- 52.3*^	95.3 +/- 67.9*
Ejection fraction during stress (%)	52.8 +/- 17.7	62.3 +/- 12.3^	32.0 +/- 12.8*	48.1 +/- 16.0*^	42.2 +/- 17.4*
End diastolic volume during stress (mL)	119.0 +/- 57.4	93.3 +/- 39.7^	173.8 +/- 50.0*	128.6 +/- 58.0*^	168.3 +/- 68.0*
End systolic volume during stress (mL)	64.2 +/- 54.4	38.7 +/- 32.4^	122.7 +/- 51.9*	72.5 +/- 53.3*^	108.0 +/- 69.9*
Left ventricular ejection fraction reserve (%)	2.0 +/- 7.4	4.2 +/- 5.8^	-3.2 +/- 7.5*	0.8 +/- 8.3*^	0.7 +/- 8.3
**Qualitative Imaging Findings**					
Sum rest score	3.0 +/- 7.1	0.2 +/- 0.8^	8.7 +/- 10.4*	5.9 +/- 9.4*	1.1 +/- 2.4^
Sum stress score	9.0 +/- 11.1	1.5 +/- 2.2^	22.5 +/- 11.5*	18.0 +/- 8.8*^	3.0 +/- 2.7^
Sum difference score	6.0 +/- 7.9	1.4 +/- 2.1^	13.8 +/- 9.2*	12.1 +/- 7.8*	1.9 +/- 2.4^
**Quantitative Imaging Findings**					
MBF at rest (ml/g/min)	0.9 +/- 0.3	1.0 +/- 0.3^	0.6 +/- 0.2*	0.9 +/- 0.3*^	0.7 +/- 0.2*
MBF during stress (ml/g/min)	1.9 +/- 1.0	2.4 +/- 0.9^	0.8 +/- 0.2*	1.8 +/- 0.7*^	0.9 +/- 0.1*
Myocardial flow reserve	2.2 +/- 0.9	2.5 +/- 0.8^	1.3 +/- 0.3*	2.2 +/- 0.8*^	1.3 +/- 0.3*

Categories with statistically significant difference (p < 0.05) from Group 1 are marked with asterisk (*) and from Group 2 with carat (^). RPP = Rate Pressure Product; MBF = Myocardial Blood Flow

Hemodynamic and structural findings of note include differences in peak heart rate, ejection fraction, wall motion abnormalities, and volumes. Both Group 2-Abnl-Qual-Abnl-Quant (abnormal qualitative, abnormal quantitative) and Group 4-Nl-Qual-Abnl-Quant (normal qualitative, abnormal quantitative) had significantly lower ejection fractions compared to Group 1-control. Group 2-Abnl-Qual-Abnl-Quant and Group 4-Nl-Qual-Abnl-Quant also had a higher proportion of patients with global wall motion abnormality at rest (84% and 50% respectively) when compared to Group 1-control (15%). Average heart volumes (end systolic volume and end diastolic volume) during both rest and stress for all three test groups were significantly higher than Group 1-control, and Group 2-Abnl-Qual-Abnl-Quant and 4 had larger volumes than Group 3-Abnl-Qual-Nl-Quant.

The 4 groups were also compared based on frequency of outcome within 1 year of testing. Table 2 depicts the percentage of patients in each group that experienced a MACE. Group 2-Abnl-Qual-Abnl-Quant (abnormal qualitative, abnormal quantitative) had a higher proportion of patients experiencing death (18.4%), MI admission (10.5%), HF admission (13.2%), when compared to Group 1-control (7.0% death, 0% MI, 0% HF). The total MACE rate was also higher for Group 2-Abnl-Qual-Abnl-Quant (40%) compared to Group 1-control (8%). In addition, Group 4-Nl-Qual-Abnl-Quant (normal qualitative, abnormal quantitative) had a significantly higher frequency of admission for HF (13%) compared to Group 1-control (0.0%). Lastly, the outcome frequencies in Group 3-Abnl-Qual-Nl-Quant (abnormal qualitative, normal quantitative) tended to be higher than Group 1-control (normal qualitative, normal quantitative), although not as high as the groups with abnormal quantitative results.

**Table 2:** Percentage of patients with 1-year outcomes clustered by normal and abnormal qualitative and quantitative results.

		Concordant Groups		Discordant Groups	
	Total	Group 1 (Nl Qual/Nl Quant)	Group 2 (Abn Qual/Abn Quant)	Group 3 (Abn Qual/Nl Quant)	Group 4 (Nl Qual/Abn Quant)
Number of patients	241	128	38	59	16
Death (%)	11.6	7.0	18.4	15.3	18.8
MI (%)	2.9	0.0^	10.5*	3.4	6.2
HF (%)	3.7	0.0^	13.2*	3.4	12.5*
PCI (%)	2.9	0.0^	13.2*	1.7^	6.2
MACE (%)	17	7.8^	39.5*	20.3*	25.0

Categories with statistically significant difference from Group 1 are marked with asterisk (*) and from Group 2 with carat (^).

### Outcome analysis

Table 3 depicts average values for several potentially confounding variables, depending on outcome status. There was no evidence of differences in average age [67.6 years (no outcome), 67.0 years (MACE outcome); p = 0.79], average BMI [30.1 (no outcome), 28.9 (MACE outcome); p = 0.23], or gender [29.25% female (no outcome), 25% female (MACE outcome); p = 0.71] between patients who had outcomes and those who did not.

**Table 3:** Average values for different imaging findings and patient factors organized by outcome along with number of outcomes.

	No Outcome	Outcome	N	p-value
**Sum Difference Score (Qualitative)**				
Death	5.4 +/- 7.8	7.3 +/- 7.0	29	0.172
MI	5.4 +/- 7.7	13.6 +/- 5.0	7	0.004*
Revascularization	5.4 +/- 7.8	12.0 +/- 5.2	7	0.015*
Heart Failure	5.6 +/- 7.8	6.6 +/- 6.5	12	0.614
All MACE	5.1 +/- 7.8	8.0 +/- 7.1	44	0.019*
**Myocardial Flow Reserve (Quantitative)**				
Death	2.21 +/- 0.88	1.86 +/- 0.72	29	0.021*
MI	2.18 +/- 0.86	1.84 +/- 1.01	7	0.413
Revascularization	2.19 +/- 0.86	1.35 +/- 0.50	7	0.004*
Heart Failure	2.20 +/- 0.87	1.65 +/- 0.68	12	0.018*
All MACE	2.25 +/- 0.87	1.82 +/- 0.75	44	0.001*
**Stress flow (ml/g/min) (Quantitative)**				
Death	1.91 +/- 0.99	1.50 +/- 0.81	29	0.016*
MI	1.89 +/- 0.98	1.06 +/- 0.53	7	0.005*
Revascularization	1.90 +/- 0.98	0.77 +/- 0.33	7	0.000*
Heart Failure	1.89 +/- 0.96	1.46 +/- 1.32	12	0.296
All MACE	1.94 +/- 0.97	1.50 +/- 0.95	44	0.006*
**BMI (kg/m^2)**				
Death	30.0 +/- 6.8	28.5 +/- 7.0	29	0.272
MI	29.8 +/- 6.8	33.1 +/- 6.6	7	0.234
Revascularization	29.8 +/- 6.8	33.0 +/- 6.0	7	0.202
Heart Failure	29.8 +/- 6.8	30.4 +/- 7.4	12	0.791
All MACE	30.1 +/- 6.9	28.8 +/- 6.5	44	0.233
**Age at time of scan (years)**				
Death	67.5 +/- 21.8	67.6 +/- 12.8	29	0.955
MI	67.4 +/- 21.2	68.9 +/- 5.5	7	0.554
Revascularization	67.4 +/- 21.2	69.5 +/- 6.1	7	0.444
Heart Failure	67.5 +/- 21.3	67.2 +/- 13.6	12	0.955
All MACE	67.6 +/- 22.4	67.0 +/- 12.2	44	0.792

Measurement variables, however, were found to significantly differ between patients who had MACE and those who did not. For example, patients who died had a significantly lower MFR than those who did not [1.86 ± 0.72 (patients who died) vs 2.21 ± 0.88 (patients who did not die); p-value = 0.021]. Significantly lower MFR was also found in patients who later underwent revascularization or had an admission for HF [1.7 ± 0.7 (patients admitted for HF), 2.2 ± 0.9 (patients not admitted for HF); p = 0.018] compared to patients who did not have events. Lastly, significantly higher SDS were found in patients who had admission for MI [13.57 ± 5.0 (patients admitted for MI), 5.40 ± 7.71 (patients not admitted for MI); p = 0.004] or underwent revascularization [12.00 ± 5.23 (patients who were revascularized), 5.44 ± 7.75 (patients who were not revascularized); p = 0.015].

### Incidence analysis

**Fig 1:**
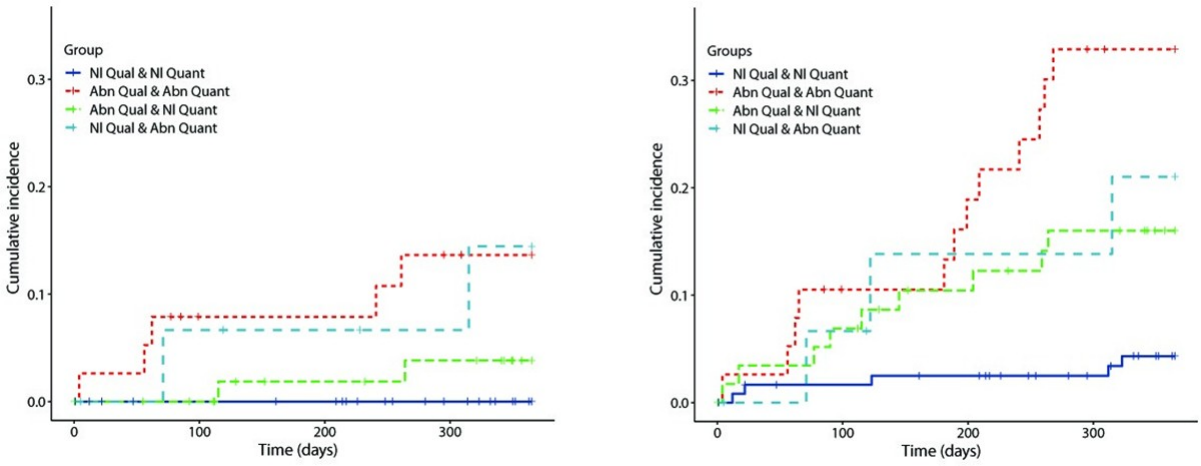
Cumulative incidence curves for HF admission (left) and composite MACE (HF admission, MI admission, revascularization, and death) (right).

Fig 1 presents cumulative incidence curves for HF admission and composite MACE. Patients in Group 2-Abnl-Qual-Abnl-Quant (abnormal qualitative, abnormal quantitative) and Group 4-Nl-Qual-Abnl-Quant (normal qualitative, abnormal quantitative) were admitted for HF earlier and more frequently than patients in Group 1-control (normal qualitative, normal quantitative) and Group 3-Abnl-Qual-Nl-Quant (abnormal qualitative, normal quantitative). S1 Table depicts hazard ratios from an unadjusted cox model analysis for PCI, HF admission, and all cause MACE. Adjustment was not performed as many of the potential covariates of interest may be independently associated with the endpoints (for example, age, hypertension, diabetes, smoking, BMI), and our intention was to demonstrate the role of imaging results in guiding management. Cox proportional hazards analysis could not be done for MI or heart failure admission with Group 1-control as reference because Group 1-control not have an event, highlighting the strongly negative predictive value of a normal qualitative, normal quantitative study. The hazard ratios (HR) for HF admission were higher in Group 2-Abnl-Qual-Abnl-Quant [HR = 4.0, 95% CI (0.8–21); p = 0.1] and Group 4-Nl-Qual-Abnl-Quant [HR = 3.9, 95% CI (0.6–28); p = 0.17] than Group 3-Abnl-Qual-Nl-Quant. Group 2-Abnl-Qual-Abnl-Quant also had total MACE earlier and more frequently compared to the other three groups, with a significantly higher HR compared to Group 1-control [HR = 5.6, 95% CI (2.4–13.2); p = 0.00007]. Group 3-Abnl-Qual-Nl-Quant and 4 also had a significantly higher risk of MACE compared to Group 1-control.

## Discussion

In this study, 31% of the included 241 PET rest/stress exams had discordance between global qualitative scores and quantitative MBF and MFR. This illustrates that discordance is fairly common. In a clinical setting, discordant findings should be reviewed with additional caution. There could be artifactual findings from a multitude of sources in both the qualitative and quantitative information. If there are no clear sources of artifacts, our findings reveal some average trends in patient risk factors and outcomes associated with different discordant categories.

There could be several modes of discordance between the visual interpretation and absolute quantification results across a range levels of severity of ischemia. In this work, we evaluate discordance of global measures of perfusion, namely the sum stress and sum difference score (to summarize the visual interpretation) and the global myocardial blood flow and flow reserve (to summarize the quantitative results). Another potential discordance is when regional information is discrepant. We defined abnormal results as having moderate to severe ischemia. Regional specific discordance and definitions of abnormality for milder degrees of ischemia were not evaluated here and likely have different prevalence levels and lead to different conclusions.

### Discordance

Patients in the discordant Group 3-Abnl-Qual-Nl-Quant tended to have some similar risk factor profiles as Group 1-control with similar BMI, hypertension, and diabetes prevalence. Group 3-Abnl-Qual-Nl-Quant did, however, show significantly larger chamber volumes and a lower EF then Group 1-control. Patients in Group 3-Abnl-Qual-Nl-Quant tended to have worse cardiovascular outcomes than Group 1-control, with a 4.0-fold increase in risk of one year MACE (p = 0.01).

The discordant Group 4-Nl-Qual-Abnl-Quant had demographic profiles suggestive of microvascular disease with a higher BMI and prevalence of hypertension and diabetes than Group 1-control. These findings support the hypothesis that this group may represent patients with diffuse perfusion abnormalities rather than a discrete flow-limiting stenosis that would appear on a qualitative perfusion image. The discordance for Group 4 could also occur in patients from three-vessel disease. This study is unable to identify the root cause of the discordance, but does find that this discordance in this patient population is associated with a higher rate of heart failure incidence than Group 1-control. And, these patients had both systolic and diastolic dysfunction compared to Group 1 potentially from microvascular disease and associated fibrosis.

Even when there are no overt signs of CAD, microvascular disease and dysfunction have been demonstrated in several conditions [29, 30], including diabetes [5, 12, 31], hypertension (presumably due to fibrosis, secondary to remodeling and inflammation [6, 32, 33]), and cigarette smoking [7, 34]. Further, microvascular disease as a clinical entity has not been well described by current clinical imaging modalities and cannot be directly visualized in vivo [19, 35]. Diagnosis and treatment of microvascular disease is further complicated when it is superimposed on epicardial CAD, often times resulting in persistent angina despite technically successful revascularization [36].

### Clinical characteristics

#### Diabetes

The discordant Group 4-Nl-Qual-Abnl-Quant as well as the concordant Group 2-Abnl-Qual-Abnl-Quant were associated with higher prevalence of diabetes. This is expected since diabetes has been linked to decreased MFR in previous studies [5, 12]. The fact that there was an increase in prevalence for both groups illustrates that normal qualitative MPI findings in a diabetic patient do not preclude the presence of microvascular dysfunction. In fact, it has been proposed that PET with quantification be used rather than SPECT when evaluating diabetic patients for suspected CAD for this reason [25].

#### Hypertension

Notably, there was no evidence of significant differences in the prevalence of hypertension between any of the three test groups and reference Group 1-control. This was unexpected because hypertension has previously been shown to be associated with reduced MFR [6, 13]. However, this may be a result of the patient population at our center. The prevalence of hypertension among adults in the United States between 2015 and 2016 was 29% [37]. Perhaps the very high prevalence of hypertension in our cohort [control group (73.9%), all other groups (73.9–83.8%)] reduced the ability to detect differences in hypertension between groups. Additionally, data on severity or duration of hypertension was not available, factors, which may influence the differences between groups.

#### Smoking

There was also no evidence of significant difference in the prevalence of smoking between any of the three test groups and Group 1-control. This was also unexpected because smoking is known to cause microvascular dysfunction and reduced MFR [7, 34]. However, perhaps the lack of a significant finding is due to the low prevalence of smoking in our study cohort (8.7–13.5%). The prevalence of smoking among adults in the US in 2015 was 15.5% [38]; whereas, the overall prevalence of smoking in the present study population was 10.9%.

Overall, this was a relatively older cohort with chronic comorbidities from a quaternary care referral center, with higher prevalence of hypertension [37] and diabetes compared to the general population [39]. However, the present study sample is representative of the population frequently referred for cardiac PET exams at tertiary and quaternary care hospitals and may be applicable in that regard.

Furthermore, because this was a cohort study, there were significant differences between the 4 groups in demographic variables such as age, BMI, and sex. Ultimately there was no apparent difference in age, BMI or sex between patients who had outcomes and those who did not. This suggests that these variables were not confounding, or at least not independently responsible for the differences observed in MACE rate between the groups.

### Limitations

This study was retrospective in nature and only included patients and exams from a single center. Likewise, the population had relatively advanced disease, with 14.8% post-heart transplant and 82.4% having had a prior cardiac catheterization, which may limit external validity. Considering outcomes were only assessed for clinical events at our institution and for a total of one-year post imaging exam, we may have missed outcomes for the minority of patients receiving follow-up care at other institutions. Furthermore, this study was based on a convenience sample of exams performed at our institution that included a majority male population (only 28.6% females). Considering the data is biased, the results are also likely biased. This type of evaluation needs to be performed on larger, unbiased populations before widespread applicability. Furthermore, our outcomes analysis was performed without controlling for other underlying comorbidities and therefore only offers insight into associations, not causality, between imaging findings and observed outcomes. Finally, this work used a broad definition of abnormal qualitative and quantitative results indicative of reversible, ischemic disease; Future work would be needed to evaluate discordance between findings reflective of irreversible, fixed defects associated with infarcted tissue.

This work used global myocardial blood flow and flow reserve as indicators for presence or absence of ischemia. While reduced global flow reserve is a known risk factor for ischemia [4, 23, 26], there could be other pathologies, independent of ischemia associated with global perfusion changes, such as atrial fibrillation that may not cause regional reductions of flow [40, 41]. This work did not perform sub-analysis to determine if the observed global perfusion problems, in the absence of qualitative ischemia, is associated with one of these other conditions. We acknowledge this limitation and recognize the definition of discordance in this work is only one of many types of discordance that could be encountered when evaluating multiple markers of ischemia.

### Value of quantification

Abnormal quantification, regardless of qualitative scores, is associated with increased risk for downstream cardiovascular outcomes. Patients who died, were revascularized, or admitted with HF had significantly lower MFRs than patients who did not have these outcomes. Past literature has supported this fact, observing higher MACE rates for patients with lower MFR [15–18].

In particular, abnormal quantification in the face of normal qualitative image findings were associated with an increased risk for HF admission and in patients with lower EF and larger myocardial volumes. We found that the frequency of discordance is not uncommon and therefore, it is important to understand the significance of the findings when the qualitative and quantitative results differ. For patients with high pretest probability for CAD (when normal qualitative scores would be unconvincing), stress PET with quantification may add value over SPECT. While further evaluation is necessary, our findings are supportive of the value of quantitative PET for symptomatic patients at risk for microvascular disease without evidence of epicardial coronary artery disease [26].

## Conclusion

In this study, we demonstrate that conflicting qualitative and quantitative findings for myocardial ischemia assessment with PET imaging are fairly common. Using a single definition based on global absolute myocardial blood flows and conventional sum-stress and difference scores, discordant findings were present in 31% of the cases in our population. Quantification supports qualitative findings when results are concordant, with the two concordant groups (abnormal vs normal) having anticipated associations with increased risk factors and outcomes. Furthermore, abnormal quantitative perfusion findings, even when discordant with qualitative perfusion assessment, were associated with an increased risk of major adverse cardiac events. Future work is needed to determine the frequency and implications of other definitions of discordance and in a larger, unbiased population to ensure appropriate use of cardiac PET.

## Supporting information

S1 Fig Clustering of patients presenting global MFR versus sum difference score. The designation of abnormal quantitative results requires both MFR<2.03 and a stress flow<1.12 ml/g/min.
(TIF)

S1 Table Hazard ratios for increased risk of PCI and MACE clustered by normal and abnormal qualitative and quantitative results. (Online table).
(DOCX)
