# Supplementary figures and images for: Patient factors and outcomes associated with discordance between quantitative and qualitative cardiac PET ischemia information
Source: PLoS One. 2021 Mar 3;16(3):e0246149. doi: 10.1371/journal.pone.0246149 (PMC7928488; doi:10.1371/journal.pone.0246149)

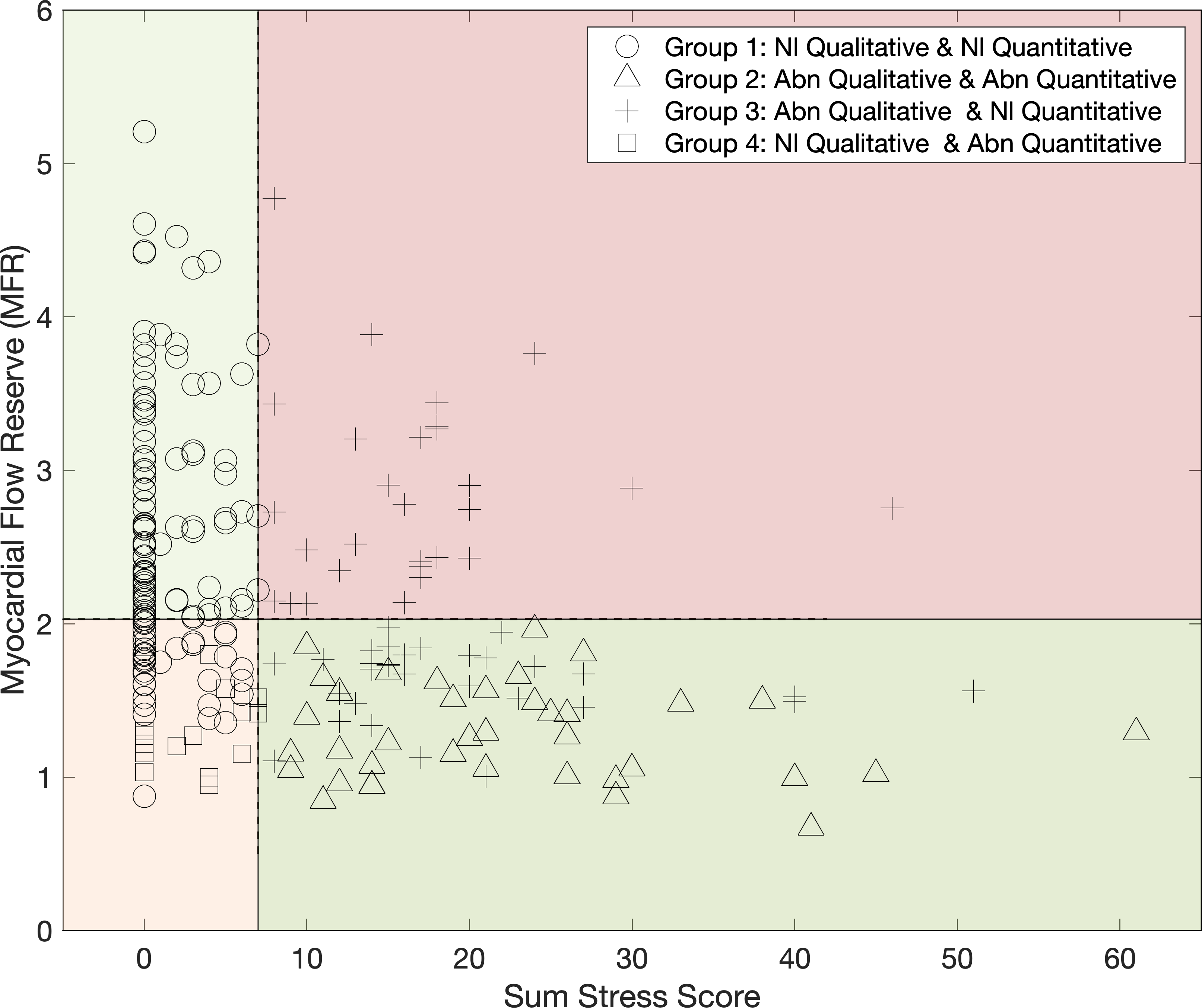

Supplement: S1 Fig — The designation of abnormal quantitative results requires both MFR<2.03 and a stress flow<1.12 ml/g/min. (TIF) [file pone.0246149.s001.tif]
