# Supplementary material for: Patient factors and outcomes associated with discordance between quantitative and qualitative cardiac PET ischemia information
Source: PLoS One. 2021 Mar 3;16(3):e0246149. doi: 10.1371/journal.pone.0246149 (PMC7928488; doi:10.1371/journal.pone.0246149)
Supplement: S1 Table — (Online table). (DOCX) [file pone.0246149.s002.docx]

S1 Table. Hazard ratios for increased risk of PCI and MACE clustered by normal and abnormal qualitative and quantitative results. (online table)

|  | Concordant Groups | | Discordant Groups | | | |  |
| --- | --- | --- | --- | --- | --- | --- | --- |
| Outcome | Group 1  (Nl Qual/Nl Quant) | Group 2  (Abn Qual/Abn Quant) | Group 3  (Abn Qual/Nl Quant) | | Group 4  (Nl Qual/Abn Quant) | | |
| PCI | 1 | 17.0 [2.0, 145.3]  *(p = 0.009*)* | 4.5 [0.4, 49.1]  *(p = 0.22)* | 8.6 [0.5, 138.2]  *(p = 0.13)* | |  |  |
| MACE | 1 | 9.0 [3.2, 25.6]  *(p = 0.0001*)* | 4.0 [1.4, 12.1]  *(p = 0.01*)* | 5.2 [1.3, 22.0]  *(p = 0.02*)* | |  |  |
| Heart Failure | NA | 4.0 [0.8, 21.0] (p=0.1) | 1 | 3.9 [0.6 – 28] (p=0.17) | |  |  |

Hazard ratios presented with [lower 95%, upper 95%] confidence intervals. Hazard ratios with statistically significant difference from reference are marked with *. Heart failure presented with Group 3 as reference considering there were no heart failure cases in Group 1 to serve as a reference.
